# P-715. Chronic Conditions as Risk Factors for Respiratory Syncytial Virus-Associated Hospitalization among Community-Dwelling Adults Aged ≥50 Years, 2017 to 2018

**DOI:** 10.1093/ofid/ofae631.911

**Published:** 2025-01-29

**Authors:** Rebecca C Woodruff, Sarah Hamid, Huong Pham, Gordana Derado, Christopher Taylor, Ahlia Sekkarie, Fleetwood Loustalot, Elizabeth Lundeen, Pam Daily Kirley, Elizabeth Austin, Lucy S Witt, Patricia A Ryan, Libby Reeg, Ruth Lynfield, Francesca Pacheco, Fiona Keating, Katherine St George, Ann Thomas, Keipp Talbot, Mary Hill, Fiona P Havers, Michael Melgar

**Affiliations:** Centers for Disease Control and Prevention, Chamblee, Georgia; Centers for Disease Control and Prevention, Chamblee, Georgia; Centers for Disease Control and Prevention, Chamblee, Georgia; CDC, Atlanta, Georgia; CDC, Atlanta, Georgia; Centers for Disease Control and Prevention, Chamblee, Georgia; Centers for Disease Control and Prevention, Chamblee, Georgia; Centers for Disease Control and Prevention, Chamblee, Georgia; California Emerging Infections Program, Oakland, California; Colorado Department of Public Health and Environment - Communicable Disease Branch, Denver, Colorado; Emory University, Atlanta, Georgia; Maryland Department of Health, Baltimore, Maryland; Michigan Department of Health and Human Services, Lansing, Michigan; Minnesota Department of Health, St. Paul, MN; University of New Mexico Health Sciences Center, Albuquerque, New Mexico; New York State Department of Health, Albany, New York; University of Rochester School of Medicine and Dentistry, Penfield, New York; Public Health Division, Oregon Health Authority, Portland, Oregon; Vanderbilt University Medical Center, Nashville, Tennessee; Salt Lake County Health Department, Salt Lake City, UT; CDC, Coronavirus and Other Respiratory Viruses Division , Atlanta, Georgia; Centers for Disease Control and Prevention, Chamblee, Georgia

## Abstract

**Background:**

In 2023, CDC recommended respiratory syncytial virus (RSV) vaccination for adults aged ≥ 60 years using shared clinical decision-making. Clinical trials of RSV vaccines in younger age groups are ongoing. Identifying chronic conditions associated with increased RSV hospitalization risk can inform vaccination policy.

**Figure 1.**
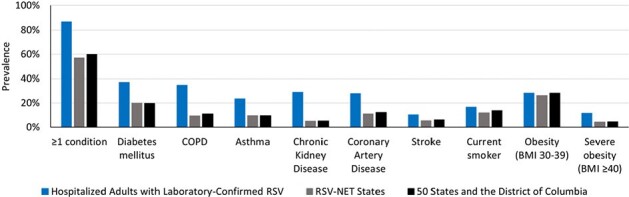
Prevalence of chronic medical conditions among community-dwelling adults aged ≥50 years hospitalized with respiratory syncytial virus (RSV),1 in RSV-NET states,2 and in 50 US states and the District of Columbia2 Abbreviations: BMI: body mass index (kg/m2), COPD: chronic obstructive pulmonary disease

1 Prevalence of chronic conditions indicated in the medical record among community-dwelling adults aged ≥50 years hospitalized with laboratory-confirmed RSV infection identified by the RSV Hospitalization Surveillance Network (RSV-NET) from October 2017 to April 2018.

2 Weighted prevalence of community-dwelling adults aged ≥50 years in RSV-NET states (CA, GA, MD, MN, NM, NY, OR, TN) or 50 states and the District of Columbia who self-reported history of chronic medical conditions on the 2018 Behavioral Risk Factor Surveillance System.

**Methods:**

We compared RSV hospitalization rates among community-dwelling adults aged ≥ 50 years with and without 9 chronic medical conditions in a 38-county catchment area across 8 states. Rate numerators included hospitalizations in adults with and without medical record documentation of each chronic condition, with laboratory-confirmed RSV infection from October 2017 to April 2018 identified by the RSV Hospitalization Surveillance Network (RSV-NET). Rate denominators were catchment area population counts of adults with and without each chronic condition estimated from the 2018 Behavioral Risk Factor Surveillance System, which relies on self-report, and the US Census. Poisson regression using Monte Carlo simulation generated adjusted rate ratios (aRR) and 95% Monte Carlo confidence intervals (CI), adjusted for age, sex, and race and ethnicity group.

**Table 1.**
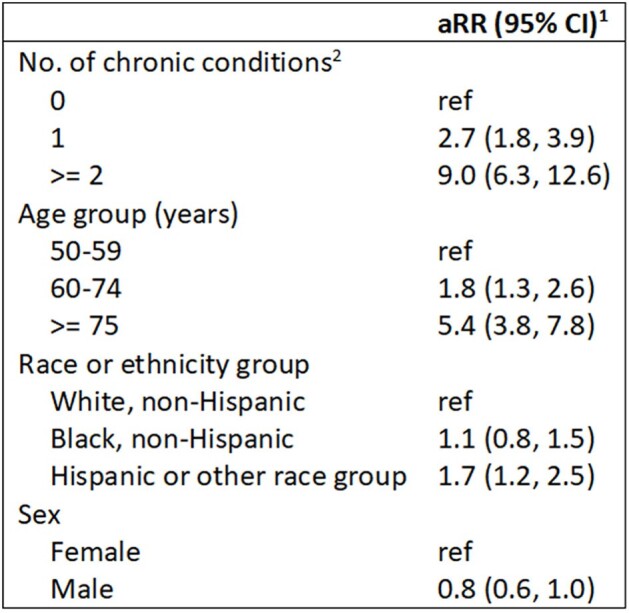
Adjusted models comparing respiratory syncytial virus (RSV) hospitalization rates among community-dwelling adults aged ≥50 years by number of chronic medical conditions— RSV Hospitalization Surveillance Network (RSV-NET), October 2017–April 2018 Abbreviations: aRR: adjusted rate ratio; CI: Monte Carlo confidence interval 1 aRR and 95% Monte Carlo CI were estimated using Poisson regression and Monte Carlo simulation after adjusting for age group (50-59 years, 60-74 years, ≥75 years), sex, and race and ethnicity group (non-Hispanic White, non-Hispanic Black, and other). 2 Includes asthma, chronic obstructive pulmonary disease, chronic kidney disease, coronary artery disease, diabetes, history of stroke, obesity (body mass index 30-39 kg/m2), severe obesity (body mass index ≥40 kg/m2), and current smoking.

**Results:**

Among 1,692 adults aged ≥ 50 years hospitalized with laboratory-confirmed RSV infection, 86.9% had ≥1 chronic condition (Figure 1). RSV hospitalization rates were higher among adults with 1 (aRR=2.7, CI: 1.8, 3.9) or ≥2 chronic conditions (aRR=9.0, CI: 6.3-12.6) vs. none and among adults aged 60-74 years (aRR=1.8, CI: 1.3, 2.6) or ≥ 75 years (aRR=5.4, CI: 3.8-7.8) vs. 50-59 years (Table 1). Adults aged ≥ 50 years with the following individual chronic conditions had higher RSV hospitalization rates compared to those without the conditions: chronic kidney disease (aRR=6.1), COPD (aRR=4.9), severe obesity (aRR=4.0), asthma (aRR=3.2), diabetes (aRR=2.5), current smoking (aRR=2.1), and non-severe obesity (aRR=1.6; Figure 2). RSV hospitalization rates among adults aged ≥ 50 years with each chronic condition were highest in the oldest age groups (Figure 3).

**Figure 2.**
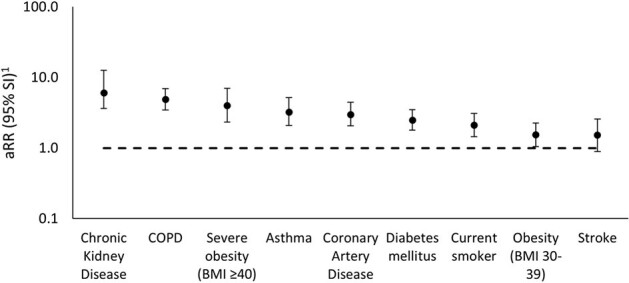
Adjusted rate ratios1 comparing respiratory syncytial virus (RSV) hospitalization rates among community-dwelling adults aged ≥50 years with and without chronic medical conditions—RSV Hospitalization Surveillance Network (RSV-NET), October 2017–April 2018 Abbreviations: aRR: adjusted rate ratio; BMI: body mass index (kg/m2), COPD: chronic obstructive pulmonary disease; SI: simulation interval

1 aRR and 95% Monte Carlo CIs were estimated using Poisson regression and Monte Carlo simulation, after adjusting for age group (50-59 years, 60-74 years, ≥75 years), sex, and race and ethnicity group (non-Hispanic White, non-Hispanic Black, and other).

**Conclusion:**

In community-dwelling adults aged ≥50 years, RSV hospitalization rates were higher among older adults and those with a history of select chronic conditions. These populations might benefit most from RSV vaccination when recommended for use.

**Figure 3.**
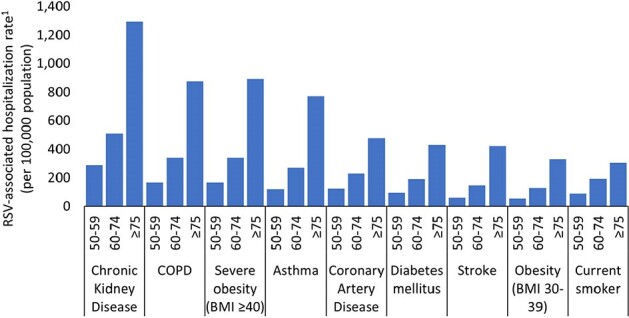
Respiratory syncytial virus (RSV) hospitalization rates1 among community-dwelling adults aged ≥50 years with chronic medical condition by age group—RSV Hospitalization Surveillance Network (RSV-NET), October 2017–April 2018 Abbreviations: BMI: body mass index, COPD: chronic obstructive pulmonary disease

1 Rates of laboratory-confirmed RSV hospitalization account for under-detection of RSV infection among hospitalized adults and sensitivity of diagnostic tests. Rates are of community-dwelling adults and exclude residents of nursing homes and long-term care facilities. Rates are not adjusted for sex or race and ethnicity group.

**Disclosures:**

**All Authors**: No reported disclosures

